# Multi-level factors linked to young adult primary care transitions: evidence from a state all-payer claims analysis

**DOI:** 10.1186/s12875-024-02463-9

**Published:** 2024-06-26

**Authors:** Sarah A. Nowak, Maija Reblin, Mark Fung, Chelsea Turley, Kirsten Threlkeld

**Affiliations:** Larner College of Medicine, University of Vermont, 89 Beaumont Ave, Burlington, VT 05405 USA

**Keywords:** Primary care, Transitions of care, Pediatric primary care, Adolescent primary care, Young adult primary care, All-payer claims, Network analysis

## Abstract

**Objective:**

Delayed transitions from pediatric to adult primary care leads to gaps in medical care. State all-payer claims data was used to assess multilevel factors associated with timely transition from pediatric to adult primary care.

**Materials and methods:**

We created a cohort of 4,320 patients aged 17–20 in 2014–2017 continuously enrolled in health insurance 36 months between 2014 and 2019 and attributed to a pediatric provider in months 1–12. We also constructed primary care provider networks identifying links between providers who saw members of the same family. Logistic regression was used to predict adult primary care in months 25–36 on family, provider, and county-level factors. Finally, we modeled the effect of county and network cluster membership on care transitions.

**Results:**

Male sex, having another family member seeing a pediatrician, and residing in a county with high pediatric care capacity or low adult primary care capacity were associated with lower odds of adult primary care transition.

**Discussion:**

We investigated factors associated with successful transitions from pediatric to adult primary care. Family ties to a pediatrician and robust county capacity to provide primary care to children were associated with non-transition to adult primary care.

**Conclusion:**

Multiple level factors contribute to non-transition to adult primary care. Understanding the factors associated with appropriate transition can help inform state and national policy.

**Supplementary Information:**

The online version contains supplementary material available at 10.1186/s12875-024-02463-9.

## Background and significance

The transition from adolescence to young adulthood is a critical and challenging period [1]. This developmental change coincides with the transition from pediatric to adult primary care, which should ideally occur when the patient is between the ages of 18-21 [2]. Adolescent readiness to transition to adult health care has increased somewhat in recent years, but remains low [3]. 

A focus on the development of adult primary care relationships for young adults is important because it serves as the foundation for care as chronic or acute health issues emerge over the life course. Further, young adults have high rates of preventable mortality [4, 5], and low rates of preventive care [6]. Despite improvements to health insurance coverage attributed to the Affordable Care Act, including the provision that allows young adults to remain on parents’ plans until age 26 [7, 8], increases in primary care utilization for young adults has been small, with past-year well-visits increasing from 28 to 31% for adults ages 18-25 [6]. 

Prior work has identified a need to adopt a social-ecological framework to understand the barriers and facilitators of young adults transitioning to adult primary care [9, 10]. Most previous research has focused on individual-level factors, such as sociodemographics or illness understanding, or health system level factors, such as availability of specialists [11, 12]. Fewer studies have focused on interpersonal factors; those that have are largely focused on the relationship between the provider, patient, and parent [10], and do not capture the broader social context. For example, network variables may provide key insight, yet are overlooked in the research literature. At the family-level, sibling or parent association with a provider, or at the practice-level, strong relationships between pediatric and adult providers, may impact the likelihood of a young adult successfully transitioning to adult care [2]. 

## Objective

Our objective was to examine factors at the individual, family, provider, and county level, that are associated with successful transition from pediatric to adult primary care.

## Materials and methods

### Data source

We used data from the Vermont Health Care Uniform Reporting and Evaluation System (VHCURES), which is a state all-payer claims database that includes Medicare, Medicaid, and private insurance, including some self-insured employer plans [13]. In total, the data capture about 60% of Vermont residents. Self-insured employer plans are not required to report data to VHCURES, but several large employers including state employee health plans voluntarily submit claims.

### Cohort definition

We created a cohort of patients ages 17–20 in 2014–2017 who were continuously enrolled in health insurance coverage for at least 36 months between 2014 and 2019 and who were attributed to a pediatric primary care provider in months 1–12. We chose this time period because the Patient Protection and Affordable Care Act (ACA) was fully enacted after 2014 and ended our analysis in 2019 to avoid having our results influenced by the COVID-19 pandemic. For each patient, the study period was the most recent continuous 36-month period they appeared in the data. Predictors were based on months 1–12 (pre-period), and outcomes were based on data from months 25–36 (post-period). We excluded 42 individuals from the analysis for whom zip code information was missing from the analysis.

### Identification of family members

We identified family members of individuals in our cohort by first identifying all insurance plan subscribers associated with insurance coverage for each member of the cohort. We then identified all individuals in VHCURES on plans with those same subscribers for a 12-month period corresponding to the pre-period for each member of the cohort. Independent variables describing family structure included in our analysis were: number of children in the family (not including the cohort member), number of young adults (ages 18–26) and number of adults (ages 27 and older). We distinguish between young adults and adults as we do not know relationships in our data set and young adults may be more likely to be siblings of the cohort member while adults may be parents or other guardians.

### Primary care attribution

We identified primary care using the Qualified Evaluation and Management (QEM) Healthcare Common Procedure Coding System (HCPCS) codes that are used by Vermont One Care for primary care attribution [14]. We then used providers’ primary taxonomy to categorized providers as general (general medicine or internal medicine), family medicine, pediatric, naturopath, or OBGYN providers. Pediatric specialists were included in the “other” category. Providers not falling into any of these categories were assigned the “other” category. We attributed patients to providers in a two-step process. We first identified any visits with a pediatrician, family medicine provider, or general provider (internal medicine providers were included in the “general” category). Patients were attributed to the provider in those categories with whom they had the majority of visits during a 12-month period. If the individual did not have any primary care visits with providers in these categories, they were attributed to the naturopath, OBGYN, or other provider with whom they had the majority of primary care visits. We attributed cohort members to a primary care provider in both the pre-period and in the post-period. We attributed family members to primary care providers in the period corresponding to the cohort members’ pre-period.

### Patient variables

In addition to the pre-period primary care provider, we constructed the following patient-level pre-period variables:

- Number of months on Medicaid (from enrollment tables).
- Number of months on commercial insurance.
- Mean age during pre-period.
- Sex.
- County (based on pre-period zip code).

### Provider variables

We constructed provider-level variables from primary care claims pulled for the entire 2014–2019 time period for each primary care provider with a physical address in Vermont to whom at least one member of either the cohort or a cohort family member was attributed. The provider variables constructed were:

- Churn, defined as the number of unique patients in each year seen by the provider who were not seen by the provider in the next consecutive year (y + 1), averaged over all years in the 2014–2019 time period. This was used as a measure of the typical duration of the provider-patient relationship in the practice.
- Panel size: mean number of unique patients seen each year.
- 95th percentile of panel member ages.

### County-level variables

We constructed two capacity measures at the county level. These were estimates of the number of primary care providers per 1,000 adults and primary care providers per 1,000 children. Adult primary care providers were those we categorized in the general medicine or OBGYN specialties. Primary care providers for children were pediatricians. We assume family medicine providers treat adult or child primary care patients in proportion to the numbers of adults and children in their respective counties.

### Outcome

Our main outcome was attribution to a non-pediatrician primary care provider in the post period.

### Descriptive analyses

We calculated the portion of the cohort with family members seeing each specialty of provider, the mean number of children, young adults, and adults identified as family members per cohort member, the mean and standard deviation of the 95th percentile of age, churn, and panel size for the provider-level variables. We also calculated the percentage of individuals in the cohort of each sex, the portion with commercial and Medicaid insurance, and the mean and standard deviation of the provider capacity measures assigned to members of the cohort. Finally, we calculated the mean and standard deviation of the network cluster measures assigned to members of the cohort.

### Provider network communities

We identified communities of primary care providers composed of pediatricians and adult primary care providers who care for members of the same family. We first identified all members of our cohort attributed to a pediatrician during the pre-period. Next, we identified all primary care providers to whom family members of those in the sample were attributed. These primary care providers were the nodes in the resulting network. Network edges were added between all pairs of providers who treated members of the same family. Using the igraph package in R, we constructed network graphs [15]. We used the cluster_louvain function from the igraph package to identify provider “communities”. Communities are groups of highly connected nodes (providers) [16]. The Louvain algorithm seeks to identify groups of nodes such that nodes are more likely to have connections with others in the same community than others in different communities. We calculated the density of each community (ratio between the number of connections among providers to the total number of possible connections in each cluster) and the assortativity (mixing between pediatricians and adult primary care providers).

### Statistical analyses

To assess the association between individual, family, provider, and county-level factors and successful transitions from pediatric to adult primary care, we conducted a logistic regression predicting receipt of adult primary care in the post-period. Independent variables were individual, family, provider, county, and network characteristics. In the supplementary material, we present a county model in which we added pre-period county membership as a predictor to the base model and a network community membership model in which we added network community membership to the base model in order to compare county and network community effects. Statistical analysis was conducted using R version 4.3.2 in RStudio 2021.09.0 for MacOS.

Patients and the public were not involved in the design, conduct, reporting, or dissemination plans of this research.

## RESULTS

Table 1 shows the summary of patient-level factors. We identified a cohort of 4,320 adolescents and young adults with a mean pre-period age of 18.4 years. 37% of the cohort had a family member attributed to a family medicine provider, 40% had a family member with a general provider, and 37% had a family member with a pediatric provider. 29% had a family member with no primary care in the pre-period. The cohort was 51% female and 49% male. 51% of cohort members had at least one month of commercial insurance in the pre-period and 58% had at least one month of Medicaid coverage in the pre-period.

**Table 1:** Characteristics of the study population. The specialty of primary care of family members is reported as the percentage of the sample with at least one family member seeing a provider in each primary care specialty. A member of the study population may be included in more than one category if different family members see providers with different specialties. Means and standard deviations for provider, county, and network measures are population-level statistics and are calculated after assigning variable values to individuals

*N*		4,320
Mean age (SD)		18.4 (1.06)
Sex	F	51%
	M	49%
Pre-period insurance	Any commercial	51%
	Any Medicaid	58%
Mean number children in family, besides self (SD)		0.48 (0.78)
Mean number of young adults in family		0.33 (0.6)
Mean number of adults in family		1.07 (0.93)
Specialty of primary care of family members	Family	37%
	General	40%
	Naturopath	2%
	OBGYN	2%
	Other	12%
	Pediatrics	37%
	None	29%
Provider-level variables	95th percentile of age	19.11 (7.68)
	Churn	0.51 (0.14)
	Panel size	998 (710)
County capacity measures	Providers per 1,000 adults	2.70 (0.74)
	Providers per 1,000 children	2.78 (0.96)
Network measures	Density	0.043 (0.066)
	Assortativity	-0.79 (0.085)

**Fig. 1:**
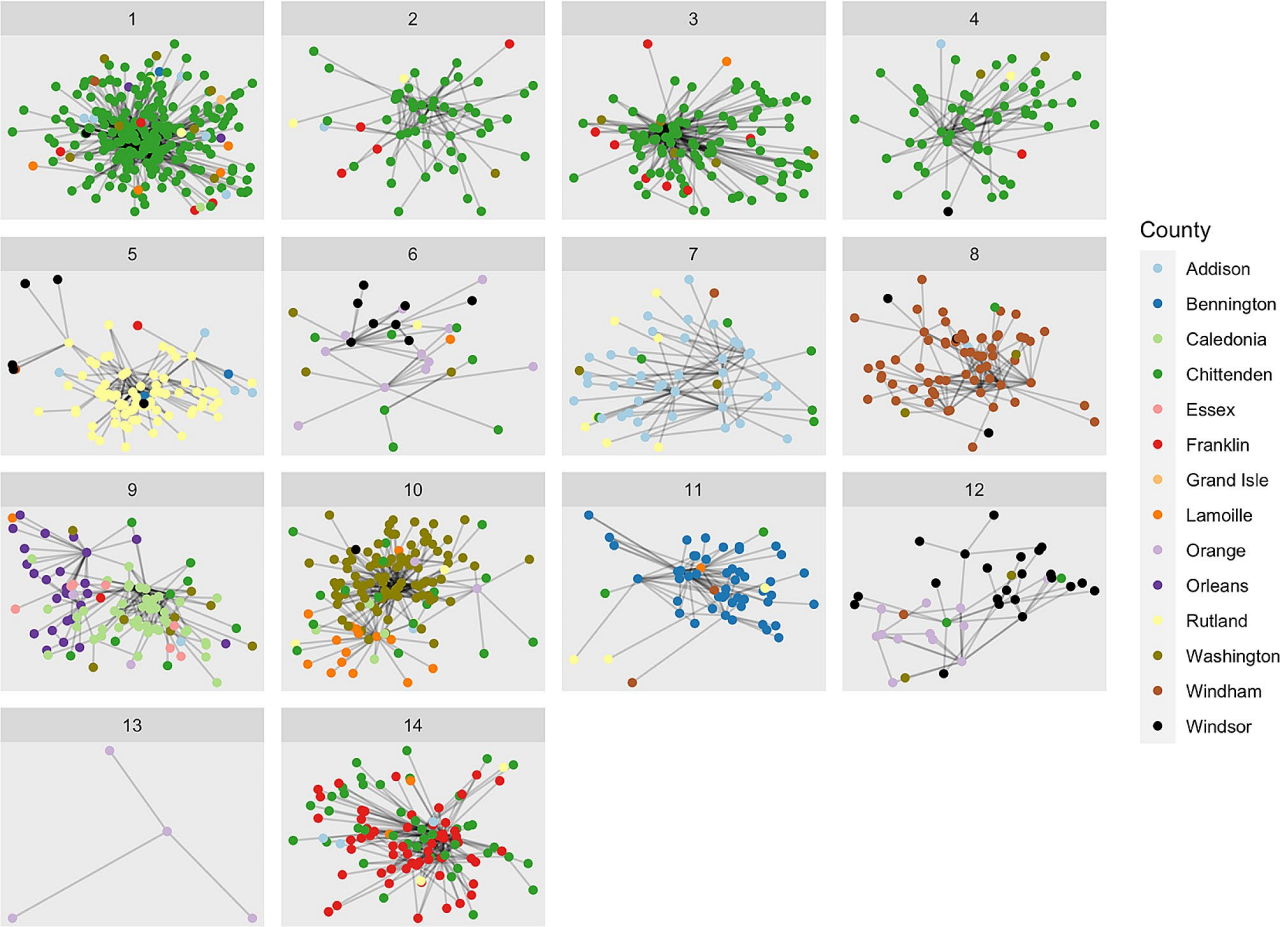
Network visualization of 14 network communities identified. Nodes (providers) have connections (edges) between them if they treat patients in the same family. Those more connected to others in the community are more central in the network visualization

Figure 1 shows the 14 largest provider network communities that we identified. Nodes (circles) represent individual primary care providers and edges (lines) indicate that the providers provide primary care for members of the same family or families. Communities 1–4 are all predominantly comprised of Chittenden county providers. Chittenden is the largest county in Vermont with nearly triple the population of the next most populous county. Communities 5, 7, 8 and 10 are primarily composed of providers from a single county. Most of the remaining communities are composed of two counties. For example, community 6 is primarily composed of providers from Orange and Windsor counties and community 9 is primarily composed of providers from Caledonia and Orleans counties.

**Fig. 2:**
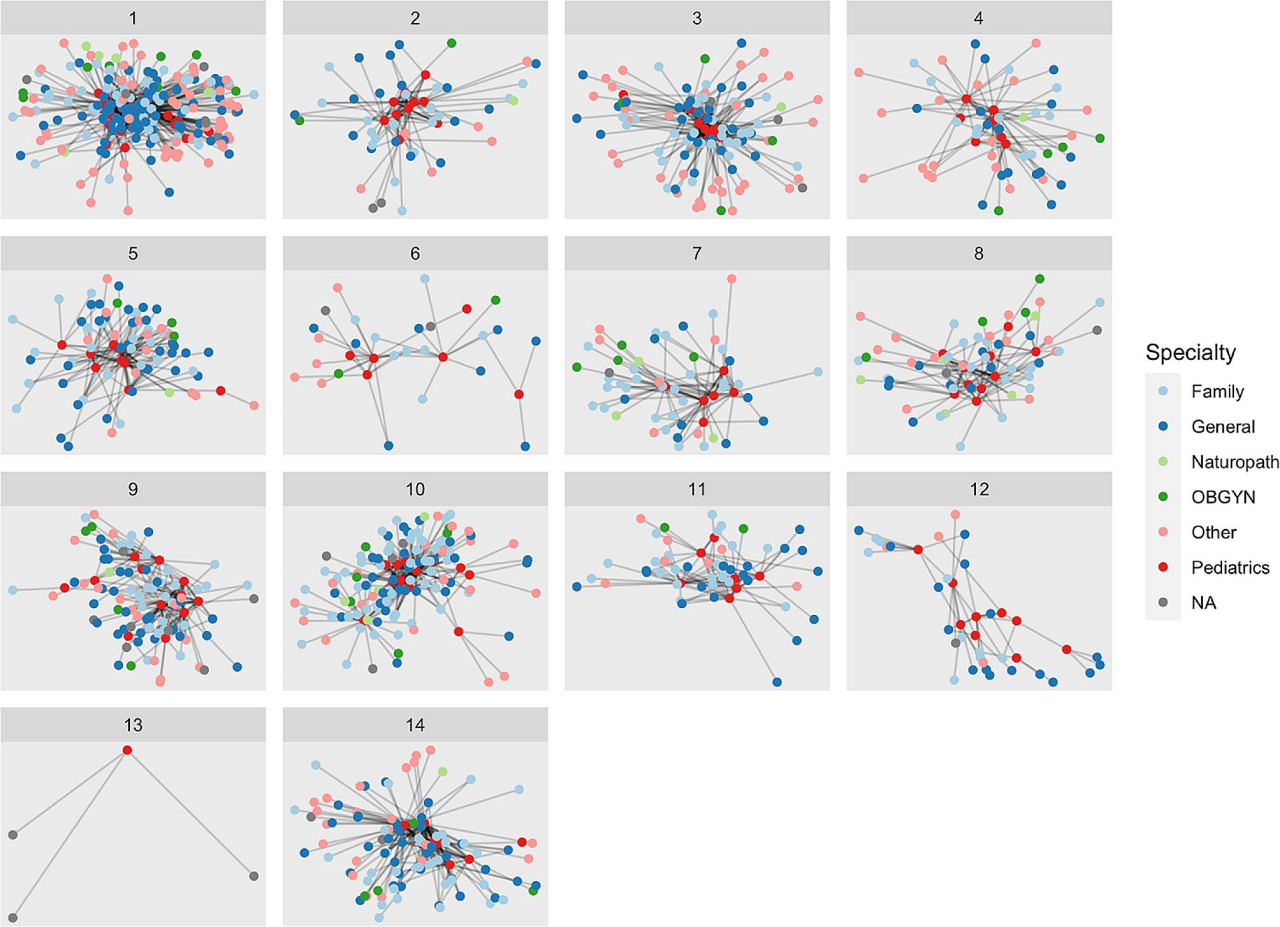
Provider network communities with colors representing primary care specialty

Figure 2 shows the same provider network communities shown in Fig. 1, but with nodes (providers) colored by provider specialty instead of by county. The network layout diagram places nodes that are more connected more central in the diagram layout while those that are less connected are on the periphery. In most network communities, the pediatric, general, and family medicine providers are more central than other, OBGYN, and naturopath providers. The mean cluster density was 0.041 (SD = 0.061). The mean assortativity in the clusters was − 0.79 (SD = 0.11).

**Table 2:** Results from an unadjusted and base model predicting transition to adult primary care among a population of adolescents and young adults with pediatric primary care. The primary care specialty of family PCP variables are binary indicators; effects are relative to not having a family member falling into each category

		Unadjusted		Base model	
		OR (95% CI)	Pr(>|t|)	OR	Pr(>|t|)
Primary care specialty of family PCP	General	0.95 (0.92,0.97)	< 0.001	1.6 (1.33,1.93)	0.044
	Pediatrics	0.91 (0.88,0.93)	< 0.001	0.96 (0.91,1)	< 0.001
	Other	0.94 (0.90,0.99)	0.024	0.93 (0.88, 0.97)	0.037
	OBGYN	0.91 (0.83,1)	0.031	0.95 (0.9,1)	0.18
	Family	0.98 (0.95,1.01)	0.79	0.93 (0.85, 1.03)	0.78
	None	1.05 (0.89,1.23)	0.020	0.99 (0.95, 1.03)	0.87
Family structure	Number of children	0.96 (0.94,0.97)	< 0.001	1 (0.97, 1.03)	0.98
	Number of young adults	1 (0.97,1.02)	0.87	1.03 (1, 1.06)	0.035
	Number of adults	0.97 (0.95,0.98)	< 0.001	1.02 (0.99, 1.05)	0.31
Provider panel characteristics (pre-period provider)	95th percentile of age	1.00 (1.00,1.00)	0.65	1.00 (1.00,1.00)	0.25
	Churn (% annual turnover)	1.23 (1.11,1.37)	< 0.001	1.37 (1.21, 1.54)	< 0.001
	Panel size (# of patients)	1.00 (1.00,1.00)	0.31	1.00 (1.00,1.00)	0.89
Individual characteristics	Sex = male (ref = female)	0.86 (0.83,0.88)	< 0.001	0.85 (0.82,0.88)	< 0.001
	Medicaid months (pre-period)	1.00 (1.00,1.01)	< 0.001	1.00 (1.00,1.00)	0.71
County capacity measures	PCPs per 1,000 adults	0.98 (0.96,0.99)	0.013	1.09 (1.04, 1.13)	< 0.001
	PCPs per 1,000 children	0.96 (0.94,0.97)	< 0.001	0.9 (0.87, 0.93)	< 0.001
Network measures	Density	1.47 (1.16,1.85)	0.0014	1.13 (0.88, 1.46)	0.34
	Assortitivity	0.99 (0.82,1.18)	0.88	1.1 (0.92, 1.33)	0.31

Table 2 shows the results of our logistic regression predicting attribution to an adult primary care provider in the post-period. Having a family member seeing a general medicine provider was associated with lower odds of transitioning to adult primary care in the unadjusted model, but associated with greater odds of transitioning to adult primary care in the adjusted model. Having another family member seeing a pediatrician was associated with lower odds of transitioning to adult primary care in both the unadjusted and adjusted models. The number of young adult family members was associated with greater likelihood of transitioning to adult primary care. Churn, the proportion of the pre-period pediatrician’s panel changing year-to-year, was associated with higher likelihood of transitioning to adult primary care in both unadjusted and adjusted models. Males were less likely to transition to adult primary care compared to females in the cohort. County capacity measures had the expected relationship with odds of transitioning to adult primary care; greater capacity to provider primary care to children was associated with lower odds of transitioning to adult primary care while higher capacity to provide primary care to adults was associated with higher odds of transitioning to adult primary care. In the unadjusted model, network density (connectedness of providers to one another) was associated with higher odds of transitioning to adult primary care, but the effect was not statistically significant in the adjusted model.

Table S1 in the supplementary material compares a model predicting transitions to adult primary care using either county or network membership as independent variables. We find that results from the network community membership model are consistent with those of the county membership model, but that the network model can offer additional nuance. For example, the community membership model distinguishes between four groups of providers in Chittenden County, the largest county in the state.

## Discussion

Our study is one of the first to focus on the transition of young adults from pediatric to adult primary care using an ecological model to guide the analysis of facilitators and barriers. As expected based on previous research, we found individual-level socio-demographic factors to be significant [3, 12]. In particular, we found that young men are less likely to transition to adult primary care in the post-period than young women. Improving transitions of care for young men may be important, as they have dramatically higher rates of preventable mortality than adolescents and young women [4]. County health system capacity measures were also found to be significant in our study. Transitions to adult primary care were associated with both robust capacity to provide primary care to adults and with constrained capacity to provide primary care to children.

Unique to our analysis, we found that family-level factors were also important to determining successful transition to adult primary care. Specifically, having a family member seeing a pediatrician was associated with not transitioning to adult primary care and having young adult family members was associated with higher odds of transitioning to adult primary care. It is possible that such relationships could be leveraged to facilitate transitioning to adult primary care. Further, there may be opportunities to ensure that pediatricians in areas with robust capacity are prepared to continue to provide primary care to young adults longer than pediatricians in other areas.

Our study is limited by the fact that the all-payer claims data is restricted to a single state, Vermont, and may not generalize to other populations. In addition, the claims data does not include information on the uninsured which is less than 3% of the Vermont population. In addition, self-insured employers are not required to contribute to the all-payer claims database although some large employers in the state, such as the University of Vermont, do contribute claims [13]. 

Further, we find that pediatricians have varied relationships with other providers. Often these are stronger for family medicine or other adult primary care providers than other specialties. However, there is some variability in these relationships. Other research has indicated that a lack of strong relationships between pediatric and adult care providers, as well as a lack of time to conduct hand-offs, can be barriers in successful referral and continued care [17–19]. 

## Conclusion

Our findings have implications for policymakers and health care systems. There is a need to develop family-centered pathways for transition to adult primary care that acknowledges existing provider capacity, while also building additional capacity to meet population needs. Further, payment models need to account for the time needed to prepare patients, for the communication required between pediatric and adult providers to ensure appropriate continuity of care.

### Electronic supplementary material

Below is the link to the electronic supplementary material.

Supplementary Material 1

## Data Availability

Data for this analysis are managed by the Green Mountain Care Board (GMCB). The study authors cannot directly share the data, based on the terms of the Data Use Agreement with the GMCB. Data can be requested from the GMCB at: https://gmcboard.vermont.gov/data-and-analytics/data-governance. The analyses, conclusions, and recommendations from the Vermont Health Care Uniform Reporting and Evaluation System (VHCURES) data are solely those of the study authors and are not necessarily those of the Green Mountain Care Board (GMCB).
