# Supplementary material for: Multi-level factors linked to young adult primary care transitions: evidence from a state all-payer claims analysis
Source: BMC Prim Care. 2024 Jun 26;25:230. doi: 10.1186/s12875-024-02463-9 (PMC11200814; doi:10.1186/s12875-024-02463-9)
Supplement: Supplementary file 1 — Supplementary Material 1 [file 12875_2024_2463_MOESM1_ESM.docx]

|  | | County model | | Network community membership model | |
| --- | --- | --- | --- | --- | --- |
|  | | OR | Pr(>\|t\|) | OR | Pr(>\|t\|) |
| Primary care specialty of family PCP | General | 0.98 | 0.052 | 0.97 | 0.11 |
|  | Pediatrics | 0.93*** | <0.001 | 0.92*** | <0.001 |
|  | Other | 0.96 | 0.11 | 0.96 | 0.12 |
|  | OBGYN | 0.94 | 0.097 | 0.94 | 0.19 |
|  | Family | 1.02 | 0.22 | 1.03 | 0.10 |
|  | none | 1.04 | 0.23 | .89 | 0.37 |
| Provider panel characteristics (pre-period provider) | 95^th^ percentile of age | 1.00 | 0.41 | 1.00 | 0.18 |
|  | Churn (% annual turnover) | 1.28*** | <0.001 | 1.24** | 0.0019 |
|  | Panel size (# of patients) | 1.00 | 0.82 | 1.00 | 0.73 |
| Individual characteristics | Gender = male | 0.85*** | <0.001 | 0.85*** | <0.001 |
|  | Medicaid months (pre-period) | 1.00 | 0.69 | 1.00 | 0.54 |
| County (comparison county is Chittenden) | Addison | 1.02 | 0.62 |  |  |
|  | Bennington | 1.11** | 0.001 |  |  |
|  | Caledonia | 1.08* | 0.027 |  |  |
|  | Essex | 1.06 | 0.52 |  |  |
|  | Franklin | 1.15*** | <0.001 |  |  |
|  | Grand Isle | 1.11 | 0.085 |  |  |
|  | Lamoille | 1.06 | 0.28 |  |  |
|  | Orange | 0.94 | 0.067 |  |  |
|  | Orleans | 0.99 | 0.89 |  |  |
|  | Rutland | 1.27*** | <0.001 |  |  |
|  | Washington | 1.16*** | <0.001 |  |  |
|  | Windham | 1.00 | 0.89 |  |  |
|  | Windsor | 1.00 | 0.91 |  |  |
| Network community membership (comparison community is 1) | 2 |  |  | 1.11** | 0.0051 |
|  | 3 |  |  | 0.98 | 0.58 |
|  | 4 |  |  | 0.97 | 0.43 |
|  | 5 |  |  | 1.31*** | <0.001 |
|  | 6 |  |  | 0.93* | 0.047 |
|  | 7 |  |  | 1.02 | 0.67 |
|  | 8 |  |  | 0.98 | 0.57 |
|  | 9 |  |  | 1.03 | 0.38 |
|  | 10 |  |  | 1.16*** | <0.001 |
|  | 11 |  |  | 1.14*** | <0.001 |
|  | 12 |  |  | 0.88 | 0.59 |
|  | 13 |  |  | 1.13 | <0.001 |
|  | 14 |  |  | 0.95 | 0.34 |

Table S1 shows the results of our logistic regressions predicting attribution to an adult primary care provider in the post-period with either county or community membership variables. In the county model, being in Bennington, Franklin, Rutland or Washington county was associated with higher odds of transitioning to adult primary care (relative to Chittenden, the largest county in the state. In the network community model, community 1, the comparison community, is a large group of providers primarily in Chittenden County. Communities 2, 5, 10 and 11 were associated with significantly higher odds of transitioning to adult primary care. Like the comparison community, community 2 is primary comprised of Chittenden County providers. Community 5 is primarily composed of Rutland providers, community 10 is primarily composed of Washington county providers, and community 11 is primarily composed of Bennington providers. See Figure 1 for community county membership. Community 6 was associated with lower odds of transitioning to adult primary care is primarily composed of providers in Windsor and Orange counties. Overall, the results of the network community model were consistent with the other models, but provided additional nuance. For example, the community algorithm identified four separate provider communities within Chittenden County (communities 1-4), and one of these (community 2) was associated with higher odds of transitioning young adults to adult primary care than the others.
